# Inhibition of microRNA‐129–2‐3p protects against refractory temporal lobe epilepsy by regulating *GABRA1*

**DOI:** 10.1002/brb3.2195

**Published:** 2021-05-24

**Authors:** Guan‐Yu Wang, Zhi‐Lin Luan, Ning‐Wei Che, De‐Bin Yan, Xiao‐Wan Sun, Cong Zhang, Jian Yin

**Affiliations:** ^1^ Department of Neurosurgery the Second Affiliated Hospital of Dalian Medical University Dalian China; ^2^ Epileptic Center of Liaoning the Second Affiliated Hospital of Dalian Medical University Dalian China; ^3^ Advanced Institute for Medical Sciences Dalian Medical University Dalian China

**Keywords:** epilepsy, GABA_A_ receptor subunit α1, *GABRA1*, hippocampus neuron, kainic acid (KA), miR‐129–2‐3p

## Abstract

**Background:**

Accumulating evidence demonstrates that certain microRNAs play critical roles in epileptogenesis. Our previous studies found microRNA (miR)‐129–2‐3p was induced in patients with refractory temporal lobe epilepsy (TLE). In this study, we aimed to explore the role of miR‐129–2‐3p in TLE pathogenesis.

**Method:**

By bioinformatics, we predicted miR‐129–2‐3p may target the gene *GABRA1* encoding the GABA type A receptor subunit alpha 1. Luciferase assay was used to investigate the regulation of miR‐129–2‐3p on *GABRA1* 3’UTR. The dynamic expression of miR‐129–2‐3p and *GABRA1* mRNA and protein levels were measured in primary hippocampal neurons and a rat kainic acid (KA)‐induced seizure model by quantitative reverse transcription‐polymerase chain reaction (qPCR), Western blotting, and immunostaining. MiR‐129–2‐3p agomir and antagomir were utilized to explore their role in determining *GABRA1* expression. The effects of targeting miR‐129–2‐3p and *GABRA1* on epilepsy were assessed by electroencephalography (EEG) and immunostaining.

**Results:**

Luciferase assay, qPCR, and Western blot results suggested *GABRA1* as a direct target of miR‐129–2‐3p. MiR‐129–2‐3p level was significantly upregulated, whereas *GABRA1* expression downregulated in KA‐treated rat primary hippocampal neurons and KA‐induced seizure model. In vivo knockdown of miR‐129–2‐3p by antagomir alleviated the seizure‐like EEG findings in accordance with the upregulation of *GABRA1*. Furthermore, the seizure‐suppressing effect of the antagomir was partly *GABRA1* dependent.

**Conclusions:**

The results suggested *GABRA1* as a target of miR‐129–2‐3p in rat primary hippocampal neurons and a rat kainic acid (KA) seizure model. Silencing of miR‐129–2‐3p exerted a seizure‐suppressing effect in rats. MiR‐129–2‐3p/*GABRA1* pathway may represent a potential target for the prevention and treatment of refractory epilepsy.

## Graphical abstract

Accumulating evidence demonstrates that certain microRNAs play critical roles in epileptogenesis. In this study, we found GABA type A receptor subunit alpha 1 (GABRA1) as a direct target of miR‐129–2‐3p, resulting in the downregulation of GABRA1 expression. Silencing of miR‐129–2‐3p exerted a seizure‐suppressing effect in KA‐induced seizure model, and miR‐129–2‐3p/GABRA1 pathway may represent a potential target of the prevention and treatment of refractory epilepsy.

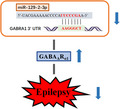

## 1 INTRODUCTION

As a common and devastating neurologic disorder, epilepsy is typical of recurring unprovoked seizures induced by abnormal firing of functional neurons in the central nervous system (CNS) (Jimenez‐Mateos & Henshall, 2013). Nowadays, about 50 million people around the world were suffering from active epilepsy with persistent seizures in a requirement for treatment, and nearly 30% of these patients are drug‐refractory (Pitkanen & Lukasiuk, 2011).

The physiological function of the CNS is manipulated by a balance of excitatory and inhibitory signaling. Experimental and clinical practice indicate several accredited pathogenetic mechanisms of epilepsy, including unbalance between excitatory (glutamate) and inhibitory (γ‐aminobutyric acid, GABA) neuronal stimulations. GABA is the major inhibitory neurotransmitter in CNS and functions via two kinds of receptors, GABA_A_ and GABA_B_ receptors. In humans, there are 19 isoforms of GABA_A_ subunits, six α, three β, three γ, and one of δ, ε, π, θ, known to form heteromeric GABA_A_ receptors and three ρ subunits that were reclassified by the Nomenclature Committee of IUPHAR (Olsen & Sieghart, 2009) to GABA_A_ from a distinct class of receptors known as GABA_C_ receptors. Genetic mutations or acquired functional alterations result in dysfunction of GABAergic neurotransmission (Simonato, 2018). Several epilepsy‐causing mutations and risk single nucleotide polymorphisms (SNPs) in GABA receptor subunits and associated proteins are related to disordered neuronal discharge, linking defective GABA inhibition with neuronal over‐excitation (Nutt & Malizia, 2001). As the major inhibitory neurotransmitter receptor in the mammalian brain, GABA_A_ receptors exist in at 20% to 50% of neuronal synapses (Nutt & Malizia, 2001). Particularly, mutations in the gene *GABRA1* which encodes GABA_A_ receptor subunit α1 have been identified as a causative factor for juvenile myoclonic epilepsies and idiopathic generalized epilepsies (Cossette et al., 2002; Hirose, 2014).

MicroRNAs are small non‐coding RNAs in the regulation of post‐transcriptional gene expression. MicroRNAs negatively regulate gene expression by targeting the 3’ untranslated region (UTR) of target mRNAs for transcriptional degradation or translational repression. These microRNAs represent a crucial layer of gene expression control in epilepsy and have a potential as therapeutic biomarkers (Shazadi et al., 2014).

Our previous study in human with temporal lobe epilepsy (TLE) indicated that miR‐129–2‐3p level was upregulated in cortical brain tissue and plasma samples from patients with refractory TLE (Sun et al., 2016). However, the specific role of miR‐129–2‐3p in temporal lobe epilepsy remains to be defined. In the present study, we aimed to further explore the underlying mechanism of miR‐129–2‐3p in refractory temporal lobe epilepsy.

## 2 METHODS AND MATERIALS

### 2.1 Cell Culture

Rat primary hippocampal neurons were cultured as previously described (Lu et al., 2016). Briefly, postnatal day 1 Sprague–Dawley (*SD*) rats were used for cell culture preparation. After meninges were removed, cerebral hippocampi were separated from the brains of 8 rats in each group. Tissue fragments were covered with dissociation medium (DMEM) with 10% FBS and dissociated by repeated aspirations via pipetting after 10‐min mild trypsinization in 0.25% Trypsin‐EDTA (Gibco, Canada) at 37 ℃. Suspension was pelleted by mild centrifugation and then seeded in culture dishes with the dissociation medium. Culture dishes were pretreated with 0.1% poly‐D‐lysine at room temperature for at least 2 hr. Cells were incubated under 5% CO_2_ at 37 ℃. After 4 hr, the medium was changed to a serum‐free medium, Neurobasal medium (Gibco, Canada) with 2% B27 supplement (Gibco, Canada), Penicillin‐Streptomycin, and 0.25% Glumax 0.5 ml. For treatment, cells were treated with a medium containing 100 μM KA for 24 hr. The human neuroblastoma cell line SH‐SY5Y was cultured and passaged in DMEM supplemented with 10% FBS, and maintained under 5% CO_2_ at 37 ℃.

### 2.2 Transfection

Transfection of cultured rat primary hippocampal neurons was performed with Nucleofector (Lonza, Basel, Switzerland) following the supplier's protocol. Briefly, cells were transfected with miR‐129–2‐3p agomir (Ago‐129), miR‐129–2‐3p agomir scrambled control microRNA (Con‐129), miR‐129–2‐3p antagomir (Anta‐129), or miR‐129–2‐3p antagomir negative control (Anta‐129 NC) (GenePharma, Shanghai, China) and seeded in a 6‐well plate (60%–80% confluency). Cells were harvested 48 hr after transfection for qPCR or Western blot analysis. *GABRA1* siRNAs were transfected in SH‐SY5Y cells by Lipofectamine® 3,000 (Thermo Fisher, MA, USA).

### 2.3 Animal Procedures

All animal care and experiments were approved by the Ethical Committee of the Dalian Medical University and in strict accordance with the National Institutes of Health guidelines for animal usage in research. Adult male Sprague–Dawley rats weighing 200–240 g (6–8 weeks) were obtained from the Medical Experimental Animal Center of Dalian Medical University. The rats were housed in a controlled temperature (21 ± 1◦C) and humidity (50%–60%) biomedical research room with a 12‐hr light/dark cycle and allowed free access to food and water available *ad libitum*. Before starting the experiments, the rats were treated to adapt to the laboratory environment for at least a week.

### 2.4 KA‐induced Seizure Model

Rats were i.c.v. injected with a microsyringe into the right lateral ventricle (Bregma coordinates: AP, 0.8 mm; L, 1.5 mm; V, 4.5 mm, based on rat brain stereotaxic atlas) of KA (40 μg/kg). Behavioral observations and seizure scoring according to the Racine scale were used to assess the activity of epilepsy. Rats with a score of 4–5 were included in the experiment. The beginning of status epilepticus (SE) was defined as the onset of continuous generalized seizure activity (stage 4 or 5 based on Racine's scale) without regaining normal behavior between seizures. Electroencephalography (EEG) was recorded at baseline and post KA injection. All the rats were monitored by EEG for up to 90 min after the successful modeling. Rats in the control group were injected with the same amount of normal saline in the right ventricle. Spontaneous recurrent seizures were monitored during the study period (6 hr, 1 day, 3 days, 7 days, and 14 days after KA treatment).

### 2.5 Intracerebroventricular Injections

The rats were anesthetized with isoflurane (5% induction and 1%–2% maintenance). MiR‐129–2‐3p antagomir (Anta) and miR‐129–2‐3p antagomir negative control (Anta NC) were, respectively, dissolved in artificial cerebrospinal fluid (Harvard Apparatus Holliston, MA, U.S.A.) at concentrations of 200 nmol/ml (1 nmol/5 μl per rat, infusion rate 0.5 μl/minute) and injected into the lateral ventricle of rats (bregma coordinates: AP, 0.8 mm; L, 1.5 mm; V, 4.5 mm, based on rat brain stereotaxic atlas) with a microsyringe. For knockdown of *GABRA1* in vivo, we designed two *GABRA1* siRNAs and the effects for these siRNAs were tested (Figure S1). We selected the most effective *GABRA1* siRNA (GR siRNA: sense sequence‐5’ GCC AGA AAU UCC CUC CCA ATT 3’ and antisense sequence‐5’ UUG GGA GGG AAU UUC UGG CTT 3’) for the following experiments. The GR siRNA was diluted in 5% glucose and then complexed with in vivo jetPEI transfection reagent (Polyplus, France) at a final concentration of 200 ng/ul. 500 ng mixture of GR siRNA and transfection reagent was injected into the lateral ventricle of rats (bregma coordinates: AP, 0.8 mm; L, 1.5 mm; V, 4.5 mm, based on rat brain stereotaxic atlas) at a rate of 0.5 μl/minute. Rats were subjected to KA treatment 24 hr after antagomir and siRNA injection. EEG was recorded 7 days after KA treatment, and the small stainless‐steel spiral electrodes were fixed in the same place for the injection. The rats were then euthanized for further molecular analysis.

### 2.6 EEG Recordings and Behavioral Observations

EEG recordings were applied as described previously (Brandt et al., 2003). Respectively, rats were implanted with three small stainless‐steel screw electrodes (diameter: 1.2 mm) into the bilateral temporal lobe and the right frontal lobe (as a reference electrode) under anesthesia with isoflurane (5% induction, 1%–2% maintenance). EEG was analyzed using Nicolet 1.0 software (Natus, USA). Seizures were defined as high‐amplitude (>29 baseline), high‐frequency (>5 Hz) poly spike discharges lasting>5s.

### 2.7 Sample preparation

Rats were euthanized by intraperitoneal injection of pentobarbital sodium and perfused with ice‐cold saline through the ascending aorta to remove intravascular blood components. Hippocampus was microdissected on wet ice for further molecular and biochemical processes. For immunostaining, deeply anesthetized rats were perfusion‐fixed through the ascending aorta with paraformaldehyde (4%).

### 2.8 Luciferase Reporter Assay

The miR‐129–2‐3p target gene was predicted by the miRDB database (http://www.mirdb.org/cgi‐bin/search.cgi). The wild‐type *GABRA1* 3’UTR sequence predicted bound by miR‐129–2‐3p were cloned into the downstream of the luciferase reporter in the pGL3‐basic luciferase vector (Promega, Madison, WI, USA). The resultant construct was designated as *GABRA1*‐WT 3’UTR Luc reporter and sequenced to validate the fragment orientation and sequence. The wild‐type 3’UTR sequence of the *GABRA1*‐WT 3’UTR Luc reporter was then mutant to construct the *GABRA1*‐MUT 3’UTR Luc reporter. The SH‐SY5Y cells were cultured and transfected with the *GABRA1*‐WT 3’UTR or *GABRA1*‐MUT 3’UTR Luc reporters for 24 hr using the Lipofectamine® 3,000 Transfection Reagent. The fluorescence was examined by using the Dual Luciferase Reporter Assay Kit (Promega, Madison, WI, USA) and a luminometer (Turner BioSystems, USA) according to the manufacturer's protocols. The results were normalized with renilla luciferase activity. The experiments of transfection were done in triplicate.

### 2.9 microRNA Expression

Total RNA was extracted using the miRcute microRNA isolation kit (Tiangen, Beijing, China), and 250 ng RNA was reverse‐transcribed using stem‐loop Multiplex primer pools (Applied Biosystems, Foster City, CA, U.S.A.). Reverse transcription (RT)‐specific primers for miR‐129–2‐3p were used for all microRNA RT. The reaction mixtures were sequentially incubated at 42 ℃ for 15 min and 85 ℃ for 5 s. Quantitative polymerase chain reaction (qPCR) was performed using a Roche Light Cycler 96 Real‐Time PCR system (Roche, Switzerland) with the following cycle: 94 ℃ for 30 s, followed by 40 cycles of 95 ℃ for 12 s and 62 ℃ for 40 s. Endogenous RNA U6 small nuclear 2 (RNU6B) was used for normalization. The relative fold change in expression of the target gene transcript was determined using the comparative cycle threshold method (2^‐△△CT^). The PCR primers for miR‐129–2‐3p were listed in **Table** **1**.

**TABLE 1:** Primers used for qPCR in this study

Gene	Sequence (5’ to 3’)	
GABRA1	Forward	GAGGGTATGCGTGGGATG
	Reverse	GCTTGACTTCTTTCGGTTCTAT
β‐actin	Forward	AGCCATGTACGTAGCCATCC
	Reverse	CTCTCAGCTGTGGTGGTGAA
miR−129–2−3p	Forward	TTCCAAGCCCTTACCCCA
	Reverse	CACTTCCTCAGCACTTGTTCCTAT
U6	Forward	GCTTCGGCAGCACATATACTAAAAT
	Reverse	CGCTTCACGAATTTGCGTGTCAT

### 2.10 Western Blot Analysis

Cells and tissues were lysed in a solution containing 20 mM Tris pH 7.5, 150 mM NaCl, 1% TritonX‐100, 2.5 mM sodium pyrophosphate, 1 mM EDTA, 1% Na_3_VO_4_, 0.5 μg/ml leupeptin, and 1 mM phenylmethanesulfonyl fluoride. Protein concentrations were determined using a BCA method (Thermo Fisher Scientific, MA, U.S.A.). Equal amount of protein from each sample was added to 10% sodium dodecyl sulfate‐polyacrylamide gel electrophoresis (SDS‐PAGE) and transfected into nitrocellulose filter membrane (NC) (Millipore, Billerica, MA, USA). Primary antibodies were incubated overnight at 4°C. Primary antibodies were as follows: GABA_A_ receptor subunit α1 (Abcam; ab33299, Cambridge, MA, USA), GAPDH (Beyotime; AF0009, Shanghai, China). After incubation, the membrane and the second anti‐incubation solution were incubated for 1 hr at room temperature. Tris‐buffered saline and Tween 20 (TBST) (PBS with 0.05% Tween 20) were used, and the membrane was washed 6 times; protein bands were analyzed by Tanon (Tanon, Shanghai, China). The images were analyzed using a Chemiluminescent Imaging System (Tanon 5,200, china).

### 2.11 Immunofluorescence Staining

GABA_A_ receptor subunit α1 immunofluorescence staining was applied by using a previous protocol (Andreska et al., 2014). Cells were washed by PBS and immobilized by 4% paraformaldehyde (PFA) followed by permeabilization with 0.2% Triton X‐100. Cells were blocked with PBS containing 10% goat serum and 1% BSA, and then incubated overnight at 4°C with primary antibody. Cells were incubated with secondary antibodies labeled with Alexa Red fluorescent dye for 1 hr, and then dyed with 4–6‐diamidino‐2‐phenylindole‐dihydrochloride (DAPI, 1:500, SLBR3299 V, Sigma, Shanghai, China) for 1 hr. Cells were observed under a Leica TCS SP8 (Leica Microsystems, Heidelberg, Germany) laser scanning fluorescence confocal microscope. Representative areas were imaged with a digital camera.

### 2.12 TUNEL Staining

TdT‐mediated dUTP Nick‐End Labeling (TUNEL) staining was performed on brain sections on Day 7 after KA treatment by using an In Situ Cell Death Detection Kit (Roche, Mannheim, Germany) according to the manufacturer's instructions. Briefly, after washing with 0.85% NaCl and PBS, the sections were fixed with 4% formaldehyde for 15 min. Following washing with PBS, the sections were covered with proteinase K solution for 15 min. After another PBS wash, the tissue sections were covered with the TUNEL reaction mixture and incubated for 1 hr. After terminating the reaction by three washes with PBS, the sections were examined and photographed using a bright field/fluorescence microscope (Leica, Wetzlar, Germany). Apoptotic cells were quantified by Image J (Rawak Software Inc., Stuttgart, Germany).

### 2.13 Statistical Analysis

Statistical analysis was performed with Prism GraphPad version 7.0 (GraphPad Software Inc., La Jolla, CA, USA) software presented as mean ± *SD*. Student's *t* test was used to access the statistical significance of the data between two groups, and the variations between the groups were examined by means of one‐way ANOVA. The staining quantitative analysis was performed by Image J software (Rawak Software Inc., Stuttgart, Germany). *p* <.05 was considered statistically significant.

## 3 RESULTS

### 3.1 miR‐129–2‐3p targets GABRA1

**FIGURE 1:**
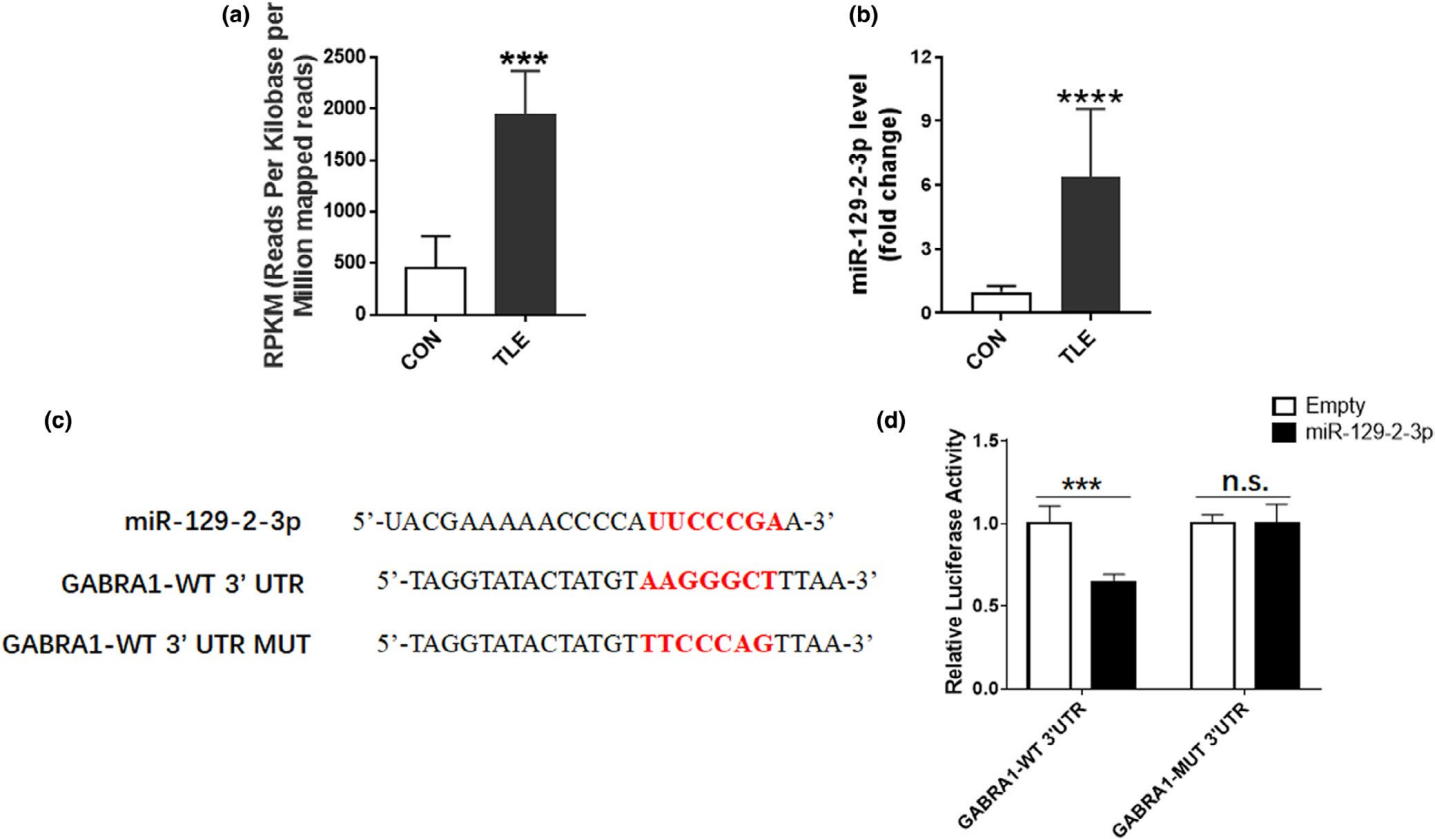
Direct target of GABRA1 by miR‐129–2‐3p (a) Quantitative measurement of miR‐129–2‐3p as assessed by RNA microarray analysis (***p <.001 compared to control group, *n* = 3 per group). (b) qPCR analysis showing the induction of miR‐129–2‐3p in refractory TLE patients (****p <.0001 compared to control group, *n* = 9 in control group and *n* = 11 in TLE group). (c) Sequences of miR‐129–2‐3p and predicted target of miR‐129–2‐3p on GABRA1 3’UTR. (d) Measured luciferase activity showing the inhibitory effect on GABRA1 3’UTR (***p <.001 compared to empty pGL3‐basic luciferase vector, *n* = 5–6 per group)

Our previous studies identified microRNAs differentially expressed in the temporal lobe of refractory TLE patients by a microRNA microarray (Sun et al., 2016), which indicated that miR‐129–2‐3p level was upregulated in cortical brain tissue and plasma of the patients (Figure 1a). To define the difference of miR‐129–2‐3p expression between the normal controls and refractory TLE patients, the miR‐129–2‐3p level was verified in a repeated sample‐set containing 9 controls and 11 TLE patients, which showed significant induction of miR‐129–2‐3p in the TLE group (Figure 1b).

To investigate the underlying mechanism of miR‐129–2‐3p in refractory TLE, miRDB (http://www.mirdb.org/cgi‐bin/search.cgi) program was referred to search for target genes of miR‐129–2‐3p. MiR‐129–2‐3p may bind to *GABRA1* 3’ UTR (Figure 1c). To confirm the regulation of miR‐129–2‐3p on the expression of *GABRA1*,a dual‐luciferase assay was applied. Consistent with the prediction, 3’UTR luciferase activity of the wild‐type *GABRA1* was markedly inhibited by miR‐129–2‐3p. However, the mutant *GABRA1* 3’UTR region has no response for miR‐129–2‐3p (Figure 1d).

**FIGURE 2:**
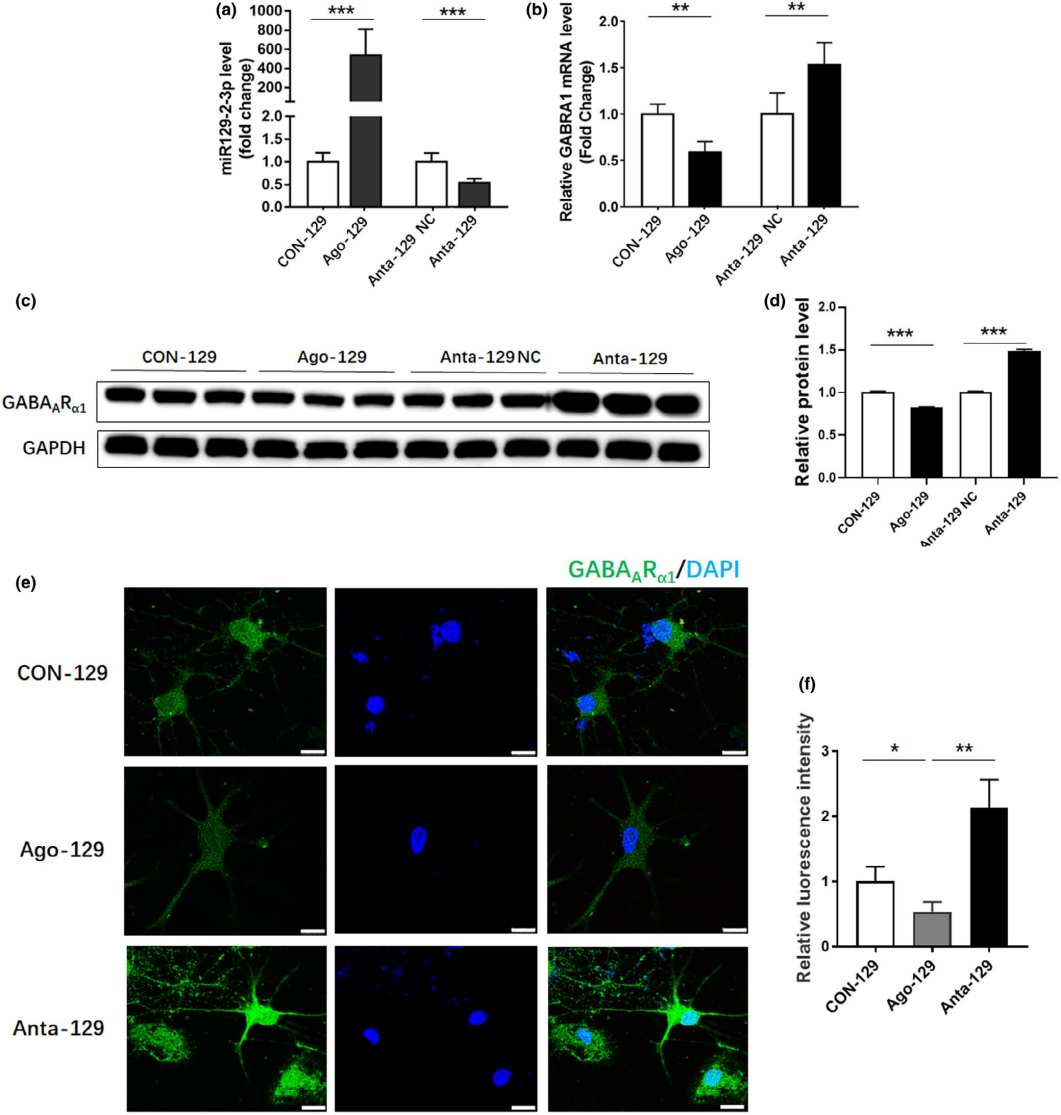
miR‐129–2‐3p regulated the expression of GABAR1 in cultured hippocampal neurons. (a‐b). qPCR analysis showing miR‐129–2‐3p (a) and *GABRA1* (b) level after treatment with miR‐129–2‐3p agomir (Ago‐129), miR‐129–2‐3p agomir scrambled control miRNA (Con‐129), miR‐129–2‐3p antagomir (Anta‐129), or miR‐129–2‐3p antagomir negative control (Anta‐129 NC) (**p <.01, ***p <.001, *n* = 4–5). (c) Western blot analysis demonstrating the GABA_A_R_α1_ protein level after treatment with Ago‐129, Con‐129, Anta‐129, or Anta‐129 NC. (d) Quantitative analysis of GABA_A_R_α1_ protein level in (c) (***p <.001 compared to the CON‐129 group, *n* = 3). (e) Immunofluorescent staining showing the sublocalization of GABA_A_R_α1_ in neuron. Bar=10 μm. (f) Quantitative analysis of the immunofluorescent signal in (e) (*p <.05, **p <.01 compared to the CON‐129 group, *n* = 3)

Transfection of mimic miR‐129–2‐3p (Ago‐129) downregulated *GABRA1* mRNA and protein levels in primary hippocampal neurons, whereas its inhibitor (Anta‐129) increased *GABRA1* mRNA and protein levels (Figure 2a‐d). In order to further determine the effect of miR‐129–2‐3p on GABA_A_ receptor subunit α1, the sublocalization of GABA_A_ receptor subunit α1 in neurons was examined by cellular immunostaining. The results showed that GABA_A_ receptor subunit α1 was broadly distributed in neurons which were upregulated by Anta‐129 (Figure 2e‐f).

### 3.2 miR‐129–2‐3p and GABA_A_ receptor subunit α1 are modulated in KA‐treated primary hippocampal neurons

**FIGURE 3:**
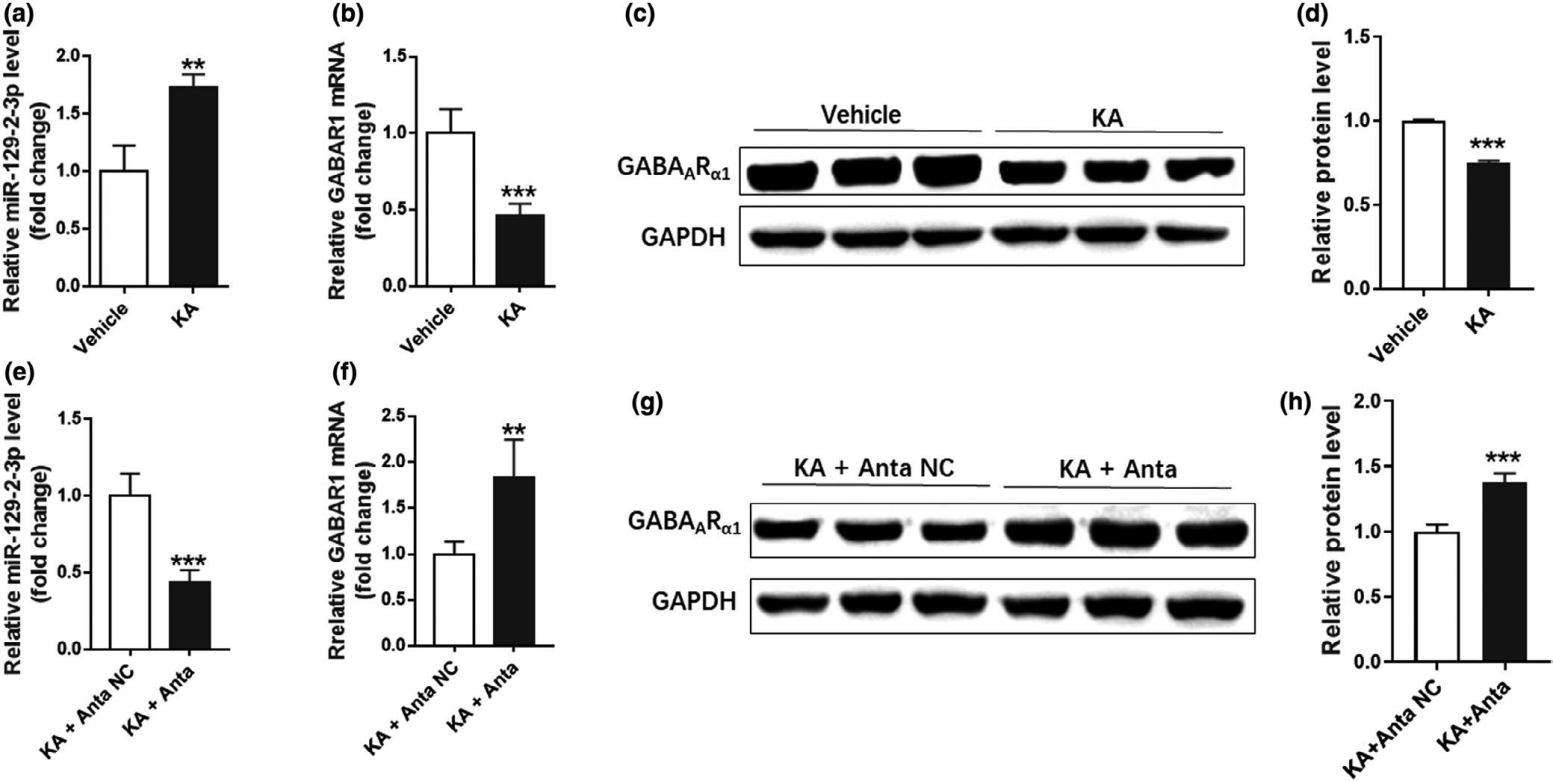
miR‐129–2‐3p was increased in KA‐treated primary hippocampal neurons. (a‐b). qPCR analysis indicating miR‐129–2‐3p (a) and *GABRA1* (b) level after KA treatment in primary hippocampal neurons (**p <.01, ***p <.001 compared to the vehicle group, *n* = 3–4). (c‐d) Western blot analysis demonstrating the GABA_A_R_α1_ protein level after KA treatment (***p <.001 compared to the Vehicle group, *n* = 3). (e‐f) qPCR analysis indicating miR‐129–2‐3p (e) and *GABRA1* (f) level after treatment of Anta‐129 together with KA (**p <.01, ***p <.001 compared to the vehicle group, *n* = 3–4). (g‐h) Western blot analysis demonstrating the GABA_A_R_α1_ protein level after treatment of Anta‐129 together with KA (***p <.001 compared to the KA+Anta NC group, *n* = 3)

After 7‐day growth of successfully cultured rat primary hippocampal neurons, 100 μM KA was added to 6‐well culture plates for 24 hr. Then, the mRNA expression levels of miR‐129–2‐3p and *GABRA1* were detected by qPCR. Western blot was used to detect the protein level of GABA_A_ receptor subunit α1. The results were as follows: miR‐129–2‐3p level was increased, and *GABRA1* mRNA expression decreased and its protein expression level was consistent with the mRNA level (Figure 3a‐d). To further determine the regulation of miR‐129–2‐3p on *GABRA1*, miR‐129–2‐3p inhibitor (Anta‐129) was transfected to the KA‐treated rat primary hippocampal neurons. The mRNA expression and protein level of GABA_A_ receptor subunit α1 were reversed after miR‐129–2‐3p inhibition (Figure 3e‐h).

### 3.3 In vivo KA‐induced seizure upregulated miR‐129–2‐3p and decreased GABA_A_ receptor subunit α1 in hippocampus

**FIGURE 4:**
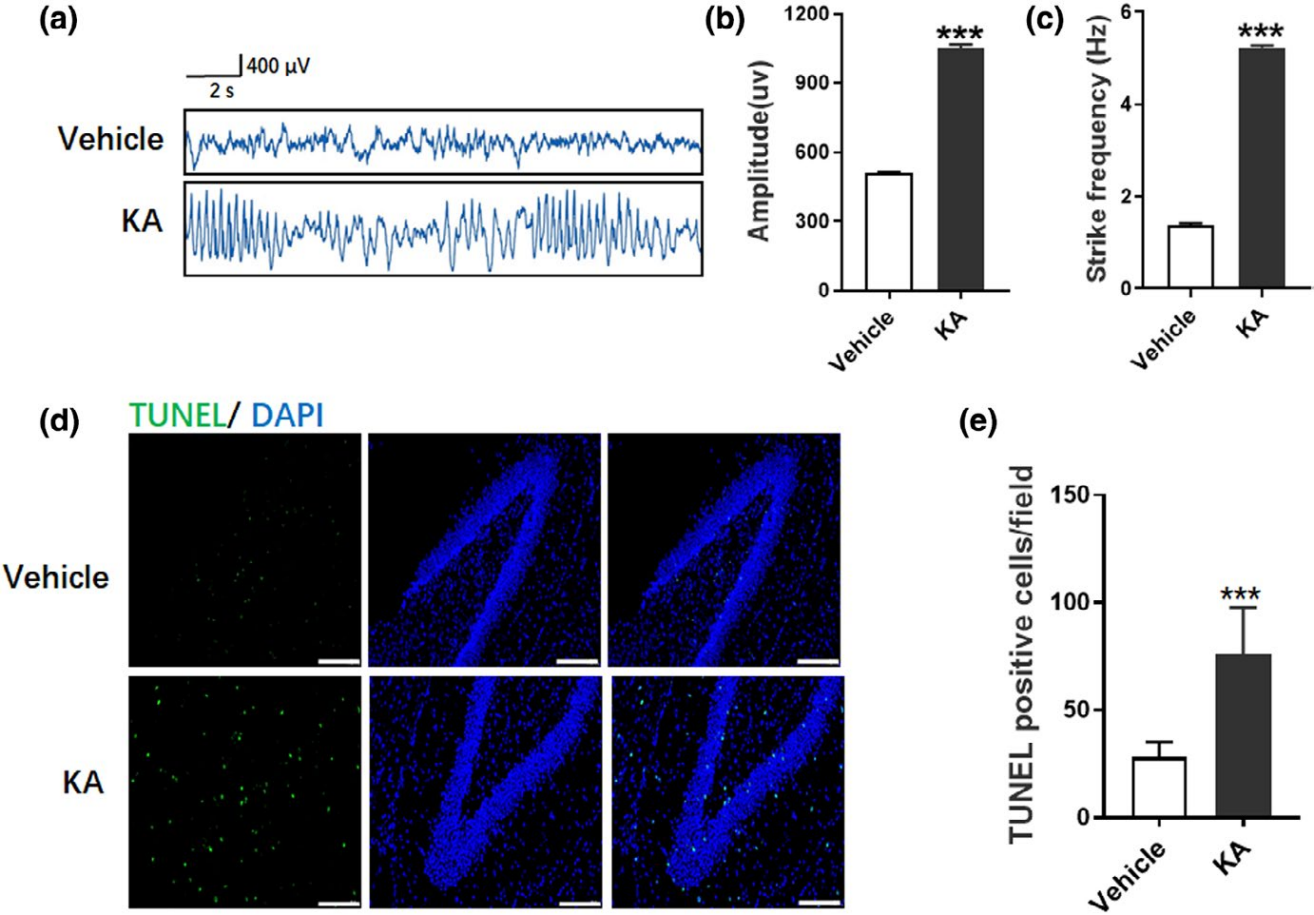
Seizure‐like EEG in KA‐induced seizure model. (a) Rats treated with vehicle and KA were analyzed by EEG and representative images were shown. (b‐c) Amplitude (b) and spike frequency (c) of seizure EEG were quantified for comparison between vehicle and KA group (***p <.001 compared to the vehicle group, *n* = 5). (d) TUNEL staining demonstrated the neuronal apoptosis in vehicle and KA group. Bar=25 μm. E. Quantitative analysis of TUNEL‐positive cells in (d) (***p <.001 compared to the Vehicle group, *n* = 3)

To study the potential involvement of miR‐129–2‐3p and GABRA1 in epilepsy, we generated an animal model of epilepsy by unilateral i.c.v. injection of KA into the *SD* rat brain. We found that rats exhibited generalized tonic‐clonic seizures after KA treatment (Figure 4a). EEG recordings at 24 hr after KA treatment identified high‐amplitude and high‐frequency discharges in rats (Figure 4b‐c). More TUNEL‐positive cells were observed after KA treatment (Figure 4d‐e), indicating KA‐induced seizure‐induced neuronal death in the hippocampus.

**FIGURE 5:**
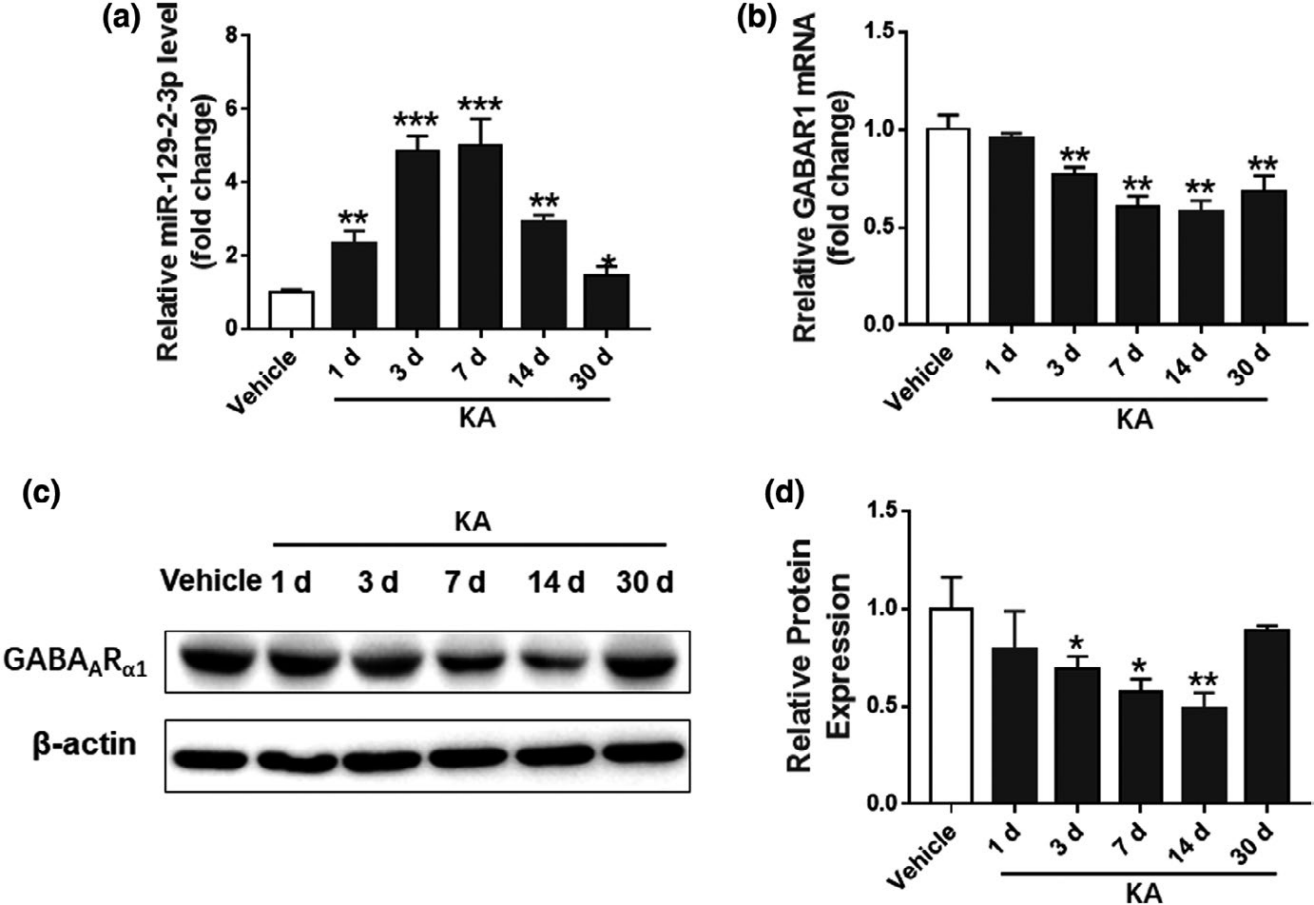
Regulation of miR‐129–2‐3p and *GABRA1* in KA‐induced seizure model. (a‐b) qPCR analysis showing the miR‐129–2‐3p (a) and *GABRA1* (b) level at different time points in KA‐induced seizure model (**p <.01, ***p <.001 compared to the vehicle group, *n* = 3–4). (c‐d) Western blot analysis demonstrating the GABA_A_R_α1_ protein level at different time points in KA‐induced seizure model (*p <.05, **p <.01 compared to the vehicle group, *n* = 3–4)

Rats in KA and control groups were sacrificed on Day 1, 3, 7, 10, or 30 after treatments, and the EEG recordings were examined to confirm the KA‐induced seizure. Levels of miR‐129–2‐3p in hippocampal regions of these rats were detected by qPCR, which showed that KA treatment resulted in an increase of miR‐129–2‐3p level and a decrease of *GABRA1* mRNA in the hippocampus on Day 1, 3, 7, and14 after treatment (Figure 5a‐b). The induction of miR‐129–2‐3p level recovered over time, and no significant difference of miR‐129–2‐3p level between KA‐treated and control rats after 14 days. Protein levels of the miR‐129–2‐3p targeted GABA_A_ receptor subunit α1 followed an opposite trend, which were remarkably lower in rat hippocampi after KA treatment. These results of GABA_A_ receptor subunit α1 were confirmed by Western blotting, which showed opposing dynamic changes to miR‐129–2‐3p expression on Day 1, 3, 7, 14, and 30 after KA treatment (Figure 5c‐d, Figure S2).

### 3.4 Inhibition of miR‐129–2‐3p alleviated seizure‐like EEG

**FIGURE 6:**
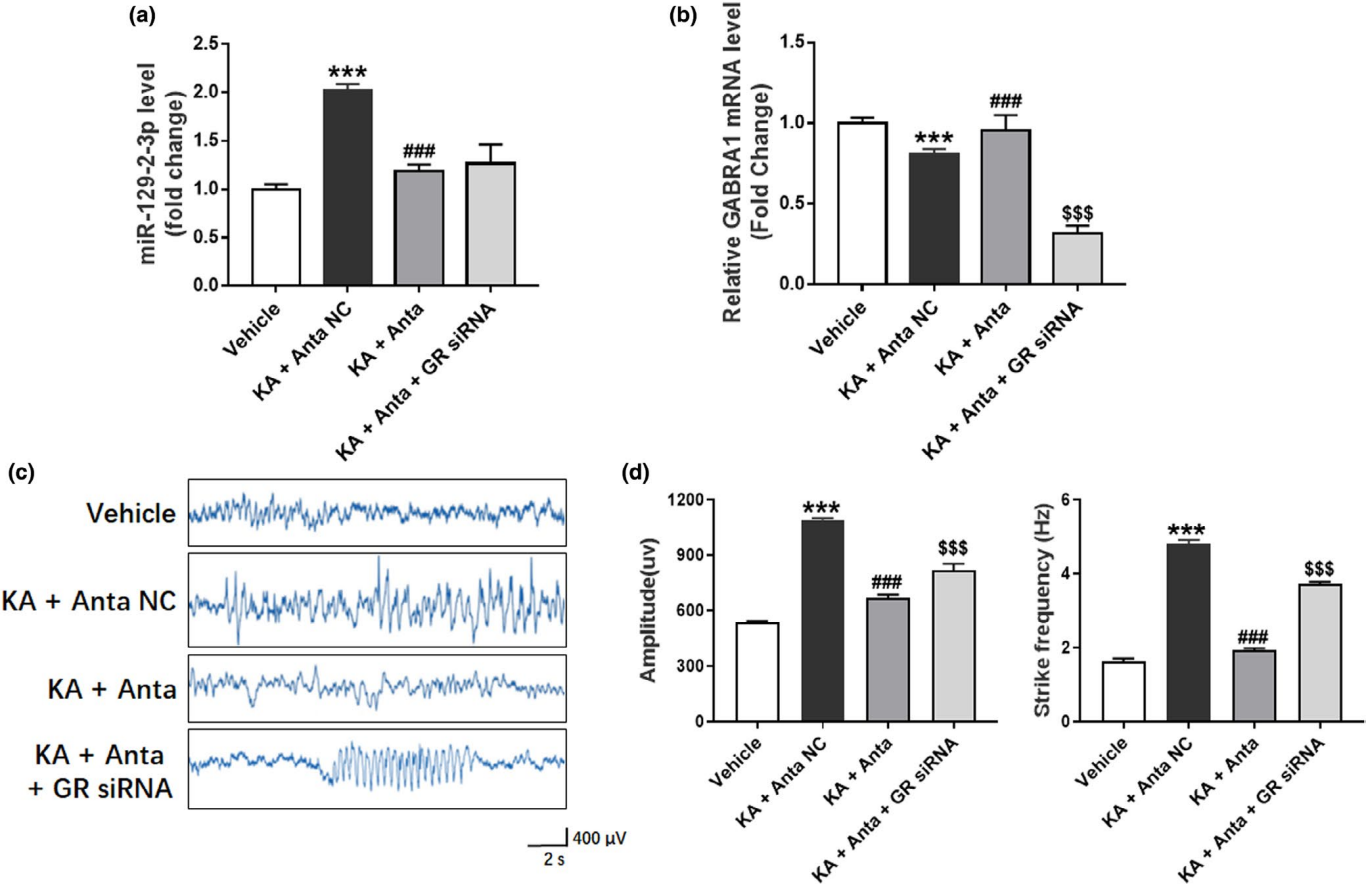
Reduced seizure‐like EEG by miR‐129–2‐3p Inhibition and counteraction of this effect by silencing of *GABRA1*. (a‐b) qPCR analysis showing miR‐129–2‐3p (a) and *GABRA1* (b) level after treatment of miR‐129–2‐3p antagomir (Anta) with or without *GABRA1* siRNA in KA‐induced seizure model (***p <.001 KA +Anta NC compared to Vehicle, ### *p* <.001 KA+Anta compared to KA +Anta NC, $$$ *p* <.001 KA +Anta + GABRA1 siRNA compared to KA +Anta, *n* = 5–10). (c) EEG and representative images for rats treated with miR‐129–2‐3p antagomir (Anta) with or without GABRA1 siRNA in KA‐induced seizure model. (d‐e) Amplitude (d) and spike frequency (e) of seizure EEG were quantified for comparison between different grouops (***p <.001 KA +Anta NC compared to Vehicle, ### *p* <.001 KA+Anta compared to KA +Anta NC, $$$ *p* <.001 KA +Anta + GABRA1 siRNA compared to KA +Anta, *n* = 5–10)

We further explored the function of miR‐129–2‐3p in vivo by inhibiting miR‐129–2‐3p expression in KA‐induced seizure model. We intracerebroventricularly injected miR‐129–2‐3p inhibitor (antagomir, Anta‐129) or its control (antagomir negative control, Anta‐129 NC) into the brains of rats, and 24 hr later the rats were treated with KA. 72 hr later, miR‐129–2‐3p level was measured which showed a significant reduction in the Anta‐129 group compared to the Anta‐129 NC group (Figure 6a), while *GABRA1* mRNA level showed a significant increase in the Anta‐129 group compared with the Anta‐129 NC group, but no compelling difference compared with the normal rats (Figure 6b). Since the level of miR‐129–2‐3p was significantly increased in KA‐induced seizure models and TLE patients, it is reasonable to speculate that its depletion might reduce seizure in KA‐induced rats. After Anta‐129 injection into the rat brain, it is worth mentioning that the severity of KA‐induced seizures in rats was significantly reduced, and abnormal EEG records were associated with high amplitude and frequency (Figure 6c‐e).

### 3.5 Silencing of *GABRA1* diminished the seizure‐suppressing effects of miR‐129–2‐3p inhibition

Meanwhile, we investigated whether epilepsy can be suppressed by the inhibition of miR‐129–2‐3p together with the silencing of *GABRA1*. Rats received *GABRA1* siRNA immediately after intracerebroventricular injection of Anta‐129 or Anta‐129 NC, followed by KA treatment 24 hr later. We measured *GABRA1* expression (72 hr after *GABRA1* siRNA treatment), and the *GABRA1* level markedly decreased (Figure 6b). The seizure‐suppressive phenotype of Anta‐129 was partly reversed in *GABRA1* siRNA cotreated rats, compared with control rats (Figure 6c‐e). Also, the apoptosis in hippocampus which was recovered by Anta‐129 disappeared after *GABRA1* silencing (Figure S3).

## 4 DISCUSSION

MicroRNAs play a major regulatory effect in gene expression. Single microRNA influences various proteins involved in diverse molecular pathways and networks. Thus, changes in level or activity of a certain microRNA may have significant effects on cellular function, indicating aberrant microRNA‐induced silencing as a striking potential disease mechanism in complex disorders. MicroRNAs have been indicated as post‐transcriptional regulators of the pathogenesis of epilepsy. Alternant microRNA level has been detected in brain and blood from patients with various epilepsy disorders. It has been reported that pilocarpine‐induced seizures induce differential regulation of microRNA‐stability related genes in rat hippocampal neurons (Kinjo et al., 2016). MiR‐146a and miR‐132 were both significantly upregulated in patients with TLE (Aronica et al., 2010; Jimenez‐Mateos et al., 2011). MiR‐324‐5p and miR‐124 were significantly reduced in an animal epilepsy model and patients with TLE (Bot et al., 2013; Brennan et al., 2016; McArdle et al., 2017). MiR‐324‐5p functions in seizure onset by targeting voltage‐gated potassium channel Kv4.2, a regulator of neuronal excitability (Gross et al., 2016). MiR‐124 attenuated the severity of epilepsy and extended the latency of epilepsy by inhibiting CREB, indicating its neuroprotective effect in epilepsy (Wang et al., 2016). In addition, epileptogenesis induced by altered microRNA signaling might lead to subsequent reorganization of hippocampal networks (Xiang et al., 2016). These data suggested that microRNAs might be effective targets for epilepsy therapy.

Our previous microRNA microarray study in patients with TLE indicated that miR‐129–2‐3p level was upregulated in cortical brain tissue and plasma samples from patients with refractory TLE (Sun et al., 2016). However, the specific role of miR‐129–2‐3p in TLE remains to be defined. Previous studies on miR‐129 mainly focused on cancer. MiR‐129–1, as a negative regulator of IGF2BP3 and MAPK1, participates in cell cycle arrest in glioblastoma and acts as a potential tumor suppressor (Kouhkan et al., 2016). The downregulation of miR‐129–1‐3p and miR‐129–2‐3p in succus gastricus of gastric cancer patients may be used as a biomarker for gastric carcinoma (Yu et al., 2013). By directly inhibiting E26 transformation specific‐1 (ETS1), miR‐129 controlled the survival, proliferation, migration, and invasion of prostate cancer cells (Xu et al., 2017). MiR‐129‐5p inhibited the development of epilepsy by inhibiting MAPK signaling and c‐Fos expression (Wu et al., 2018).

To further explore the underlying mechanism of miR‐129–2‐3p in refractory temporal lobe epilepsy, miRDB (http://www.mirdb.org/cgi‐bin/search.cgi) program was applied to search for target genes of miR‐129–2‐3p. We found that *GABRA1* 3’ UTR may be directly anchored by miR‐129–2‐3p. In common, microRNAs negatively regulate the expression of a gene by binding to its 3′‐UTR. We proved that the regulation of *GABRA1* by miR‐129–2‐3p by dual‐luciferase reporter assay, indicating that *GABRA1* 3’ UTR may be directly anchored by miR‐129–2‐3p. As the main inhibitory neurotransmitter in mammal, γ‐aminobutyric acid (GABA) is widely distributed in the whole nervous system (Brooks et al., 2015). It has been acknowledged that epilepsy is related to the decline of inhibitory synaptic function, and stimulating GABA‐mediated inhibitory synapse can effectively alleviate the epileptic attack (Schipper et al., 2016). The major inhibitory ligand‐gated channels in mammalian brain are GABA_A_ receptors, and approximately 60% of GABA_A_ receptors are α1‐containing GABA_A_ receptors (Benarroch, 2007). The gene encoding GABA_A_ receptor subunit α1 isoform is *GABRA1* on chromosome 5q34 in human. Heterozygous *GABRA1* knockout mice and *GABRA1* ^A322D^ knockin mice experienced absence‐like seizures accompanied with EEG spike‐wave discharges and developed myoclonic seizures late in life (Arain et al., 2012). In clinical research, a mutation of *GABRA1* was observed in children with absence seizure (Maljevic et al., 2006). Mutation in *GABRA1* has also been found in affected individuals of a large French Canadian family with juvenile myoclonic epilepsy (Cossette et al., 2002). Accumulated evidence indicated that mutations in *GABRA1* contribute to the genetic etiology of both benign and severe epilepsy syndromes (Johannesen et al., 2016). Many of the currently used antiepileptic drugs act through enhancing GABAergic functions that include positive allosteric modulators of GABA_A_ receptors, blockers of GABA uptake, and blockers of GABA degradation (Khazipov et al., 2015).

In recent years, cholesterol‐binding antagomirs and agomirs were used to inhibit or enhance the targeted microRNAs (Krutzfeldt et al., 2005). We found that miR‐129–2‐3p was significantly higher in Ago‐129 (agomir) group than that in Con‐129 (agomir scrambled control microRNA) group, while miR‐129–2‐3p significantly downregulated in Anta‐129 (antagomir) group than that in Anta‐129 NC (antagomir negative control) group. However, the alteration of *GABRA1* expression level was contrary to the miR‐129–2‐3p level. KA‐induced seizure model is a widely used and recognized model for refractory TLE (Lauren et al., 2010). We found that, after treated with KA, miR‐129–2‐3p increased significantly in primary hippocampal neurons, and the levels of *GABRA1* decreased markedly. However, Anta‐129 reversed the alteration of miR‐129–2‐3p level and *GABRA1* expression, which further demonstrated that miR‐129–2‐3p may regulate *GABRA1* and act as a therapeutic target. In addition, we found that the mRNA and protein expression of *GABRA1* decreased gradually within 14 days after KA induction in rats, which is consistent with previous research (Lauren et al., 2005). We also compared the EEG and immunostaining of the KA‐treated rats at different time points, which showed a negative correlation with the *GABRA1* expression, especially on Day 7. Based on the above results, we cotreated rats with KA and Anta‐129 (antagomir) and observed the *GABRA1* level and EEG, which showed an over‐expression effect on *GABRA1* and effective inhibition on EEG. However, when *GABRA1* interfered by siRNA in hippocampus, the protective function of Anta‐129 was eliminated, which further indicated the involvement of miR‐129–2‐3p in epilepsy was related to its regulation on *GABRA1*.

Neuronal cell death is a direct pathophysiological consequence of various brain injuries caused by epilepsy. Epileptic attack is associated with acute and delayed neuronal death, gliosis, changes in synaptic and circuit functions, neuroinflammation, neurodysplasia, and extracellular matrix remodeling (Henshall & Engel, 2013). Loss of neurons in the hippocampus is a common pathological marker for cerebral ischemia in human and aminol models. Neuronal death may promote hyperactivity by reactive gliosis and inflammation accompanied by neuronal damage (Maroso et al., 2010). To determine whether miR‐129–2‐3p participates in the process of neuron apoptosis, we investigated neuronal apoptosis by TUNEL staining. The apoptotic effect of KA for hippocampal neurons was reversed by Anta‐129 (antagomir), while the anti‐apoptotic role of Anta‐129 (antagomir) was weakened after *GABRA1* knockdown by siRNA. These data indicated that miR‐129–2‐3p may be involved in the regulation of apoptosis. Inhibition of miR‐129–2‐3p has a certain neuroprotective effect. However, in the absence of *GABRA1*, the anti‐apoptotic role of miR‐129–2‐3p was counteracted.

The results of the current study demonstrated that miR‐129–2‐3p may be involved in the mechanism of refractory epilepsy by regulating *GABRA1*, and a miR‐129–2‐3p antagomir alleviated seizures and abnormal EEG findings. We therefore identified miR‐129–2‐3p as a novel seizure regulator and characterized the miR‐129–2‐3p/GABRA1 pathway as a potential target in the prevention and treatment of epilepsy.

## CONFLICT OF INTEREST

All authors claim that there are no conflicts of interest.

## AUTHOR CONTRIBUTIONS

Guan‐Yu Wang: Methodology, Software, Formal analysis, and Writing—original draft. Zhi‐Lin Luan: Conceptualization, Methodology, Data curation, Validation, Writing—review & editing, and Funding acquisition. Ning‐Wei Che: Methodology and Formal analysis. De‐Bin Yan: Formal analysis and Software. Jian Yin: Conceptualization, Supervision, Writing—review & editing, and Funding acquisition.

### PEER REVIEW

The peer review history for this article is available at https://publons.com/publon/10.1002/brb3.2195.

## Supporting information

Figure S1‐S3

## Data Availability

The data that support the findings of this study are available from the corresponding author upon reasonable request.
