# Supplementary material for: Inhibition of microRNA‐129–2‐3p protects against refractory temporal lobe epilepsy by regulating GABRA1
Source: Brain Behav. 2021 May 24;11(7):e02195. doi: 10.1002/brb3.2195 (PMC8323041; doi:10.1002/brb3.2195)
Supplement: Supplementary file 1 — Figure S1‐S3 [file BRB3-11-e02195-s001.docx]

**
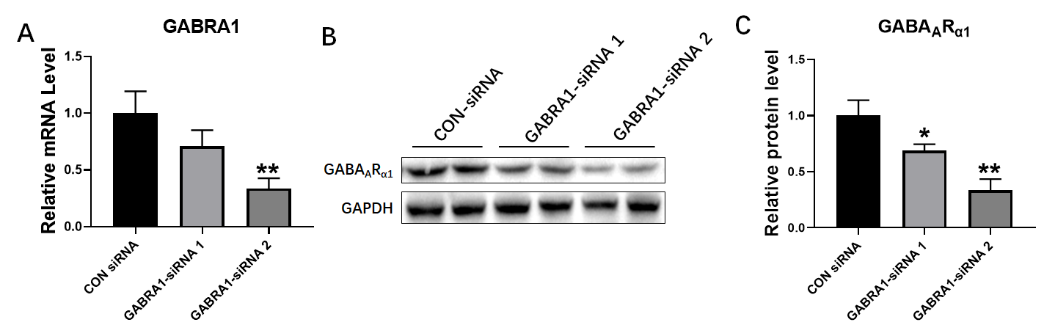
Figure S1. The knockdown effects of *GABRA1* siRNAs**

**(A)** qPCR analysis showing the knockdown effect of two *GABRA1* siRNAs on the GABRA1 mRNA level (***p* < 0.01 compared to the CON-siRNA group, n=3). **(B)** Western blot analysis demonstrating the GABA_A_R_α1_ protein level after siRNA treatment; **(C)** Quantitative analysis of GABA_A_R_α1_ protein level in (B) (**p* < 0.05, ***p* < 0.01 compared to the CON-siRNA group, n=3).

**
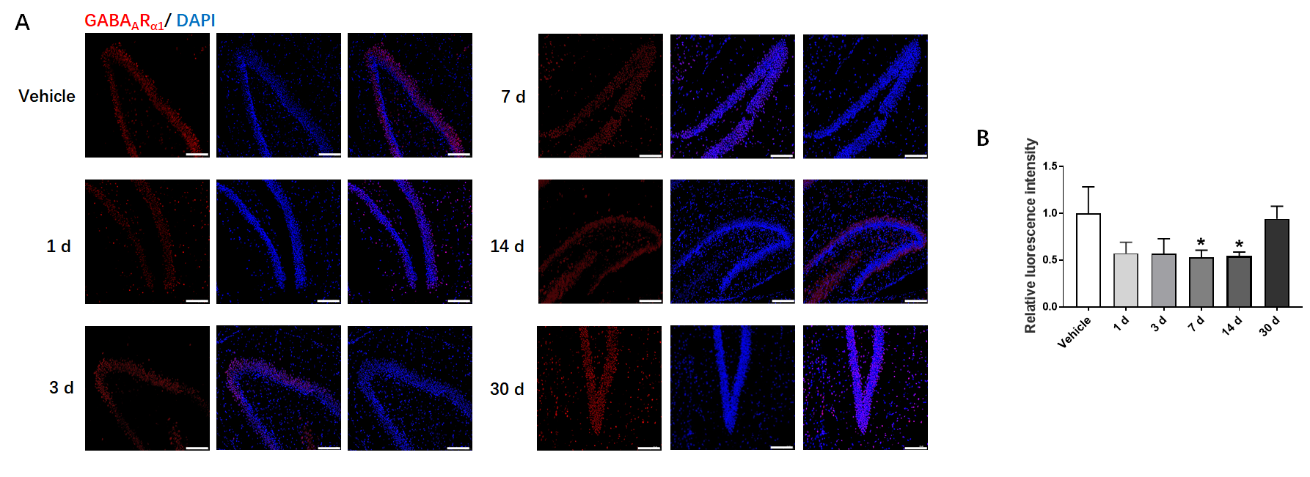
**

**Figure S2. Immunofluorescence staining of GABA_A_R_α1_ at different time points after KA treatment.**

**(A)** Presentative images for the immunofluorescence analysis of GABA_A_R_α1_ protein level after KA treatment. Bar=25 μm. (**B**) Quantitative analysis of GABA_A_R_α1_ protein level in (A) (**p* < 0.05, ***p* < 0.01 compared to the Vehicle group, n=3).

**
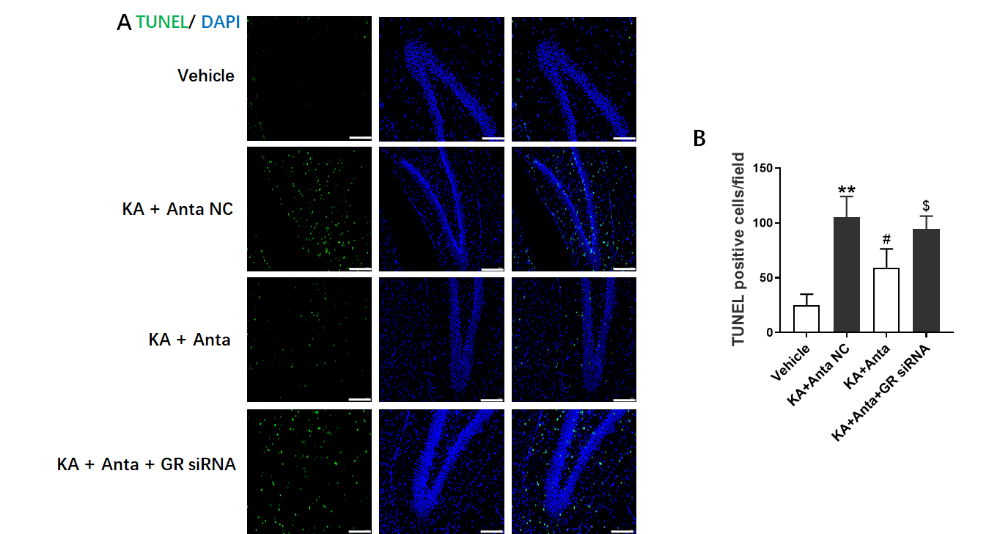
**

**Figure S3. Neuronal death inhibited by miR-129-2-3p inhibition was reversed by *GABRA1* silencing in hippocampus**

**(A)** Presentative images for the TUNEL analysis of apoptotic cells after different treatments. Bar=25 μm. (**B**) Quantitative analysis of TUNEL-positive cells in **(A)** (***p* < 0.01 KA + Anta NC compared to Vehicle, # *p* < 0.05 KA+ Anta compared to KA + Anta NC, $ *p* < 0.05 KA + Anta + GABRA1 siRNA compared to KA + Anta, n=3).
